# Resistance to Erythropoiesis-Stimulating Agents Is Associated with Arterial Microcalcification in Early Hemodialysis Patients

**DOI:** 10.1155/2014/731296

**Published:** 2014-03-17

**Authors:** Hye Sung Won, Su Jin Choi, Yu Seon Yun, Ok-Ran Shin, Yoon Ho Ko, Young Soo Kim, Sun Ae Yoon, Young Ok Kim

**Affiliations:** ^1^Department of Internal Medicine, College of Medicine, The Catholic University of Korea, Seoul, Republic of Korea; ^2^Department of Pathology, College of Medicine, The Catholic University of Korea, Seoul, Republic of Korea; ^3^Division of Nephrology, Department of Internal Medicine, Uijeongbu St. Mary's Hospital, College of Medicine, The Catholic University of Korea, 271 Cheon bo-ro, Uijeongbu-si, Gyeonggi-do 480-717, Republic of Korea

## Abstract

The aim of this study was to evaluate the relationship between arterial microcalcification (AMiC) and erythropoiesis-stimulating agents (ESA) hyporesponsiveness in hemodialysis patients. The presence of AMiC was confirmed by pathologic examination of von Kossa-stained arterial specimens acquired during vascular access surgery. We assessed the ESA hyporesponsiveness index (EHRI), defined as the weekly ESA dose per kilogram body weight divided by the hemoglobin level. AMiC was detected in 33 (40.2%) of 82 patients. Patients with diabetes had a higher incidence of AMiC than patients without diabetes. The serum levels of albumin and cholesterol were higher in patients without AMiC than in patients with AMiC. The serum levels of intact parathyroid hormone were lower in patients with AMiC than in patients without AMiC. The serum levels of phosphate and calcium-phosphorus product did not differ between the two groups. The mean EHRI value was higher in patients with AMiC than in patients without AMiC. In multivariate analyses, ESA hyporesponsiveness and diabetes showed a significant association with AMiC. In conclusion, ESA hyporesponsiveness may be a clinical relevant parameters related to AMiC in hemodialysis patients.

## 1. Introduction

Anemia and vascular calcification are common complications in hemodialysis (HD) patients, both of which are independently associated with an increased risk of cardiovascular mortality [1, 2]. Anemia in patients with chronic kidney disease (CKD) is usually corrected with erythropoiesis-stimulating agents (ESAs), but some patients experience ESA hyporesponsiveness, defined as a failure to achieve target hemoglobin/hematocrit levels despite a higher than usual dose of ESA [3]. The Kidney Disease Outcomes Quality Initiative (KDOQI) guidelines define ESA hyporesponsiveness as a continued need for more than 300 IU/kg per week recombinant human erythropoietin to maintain adequate hemoglobin levels [4]. Uremic patients have inflammation and oxidative stress caused by various patient or dialysis-related factors [5, 6]. This inflammation has been reported to be an important factor in ESA hyporesponsiveness. We previously reported that the level of the proinflammatory cytokine interleukin-6 (IL-6) is a strong predictor of ESA hyporesponsiveness [7].

Arterial calcification can occur in both intimal and medial layers, and two types of calcifications have different pathophysiology. Intimal calcification represents an advanced stage of atherosclerosis and medial calcification can lead to reduced compliance due to arterial stiffening resulting in an impaired vasodilation during ischemia and a higher risk of cardiovascular mortality. Medial calcification is associated with diabetes and CKD [2, 8]. The prevalence of vascular calcification is higher in patients with CKD than in the general population. Previous studies showed that 40% of patients with CKD showed coronary arterial calcification compared with 13% of control subjects of similar age with no renal impairment [9]. The causes of vascular calcification in patients with CKD are multifactorial. Although previous studies have focused primarily on abnormalities in mineral and phosphate metabolism, recent studies have shown that inflammation and oxidative stress could contribute to the development of vascular calcification [8, 10, 11].

Based on these common roles of inflammation and oxidative stress in the pathogenesis of ESA hyporesponsiveness and vascular calcification, we aimed to evaluate the relationship between arterial medial microcalcification (AMiC) and ESA hyporesponsiveness in HD patients.

## 2. Materials and Methods

### 2.1. Patients

We identified the presence of vascular calcification by histological examination. Eighty-nine patients who received vascular access surgery between September 2010 and November 2012 in Uijeongbu St. Mary's Hospital were evaluated, and thus they just started HD or prepared to start HD. We included patients who received ESAs for more than 3 months. The followings were excluded: (1) patients who had experienced overt inflammation at the time of evaluation; (2) patients who had other malignant diseases; (3) patients who had a recent blood transfusion; and (4) patients who had an iron deficiency, based on their transferrin saturation (TSAT) and ferritin levels. Iron deficiency was defined as a serum ferritin level of <200 ng/mL or TSAT <20%. Finally, 82 patients were included in this study. The study was approved by the ethics committee of the Institutional Review Board of The Catholic University of Korea, Uijeongbu St. Mary's Hospital.

### 2.2. ESA Hyporesponsiveness and Laboratory Measurements

The ESAs darbepoetin or epoetin was administered subcutaneously at the end of dialysis. The target hemoglobin level was 11 g/dL. The initial epoetin dose was 60–120 IU/kg per week in two to three doses per week, and the darbepoetin dose was 0.45 *μ*g/kg per week given once a week. A ratio of 1 : 200 was used to convert darbepoetin to the equivalent epoetin dose. ESA doses were recorded over 3 months from 1 month before vascular access surgery, and the mean values were used in this study. We calculated the ESA hyporesponsiveness index (EHRI), defined as the weekly ESA dose per kilogram of body weight divided by the hemoglobin level (g/dL). Thus, higher EHRI values mean a reduced response to ESAs. Patients with iron deficiency received parenteral iron at a dose of 100 mg/week until the target ferritin and TSAT levels were achieved, and then they took 512 mg of oral ferrous sulfate (160 mg of elemental iron) per day. If the serum ferritin level was >800 ng/mL and/or TSAT was >50%, iron therapy was stopped.

Laboratory parameters were used as mean values measured over 3 months from 1 month before vascular access surgery. The calcium-phosphorus product (CaxP) was determined by multiplying serum calcium and phosphorus levels. The following clinical data were collected: age, sex, body mass index, causes of CKD, Kt/V, normalized protein catabolic rate, medical history, duration of HD, and medications.

### 2.3. Arterial Microcalcification

We previously reported the assessment of AMiC in HD patients [12]. In brief, the presence of AMiC was assessed in arterial specimens acquired during vascular access surgery. The 5 mm diameter, ellipse-shaped arterial specimens were obtained from the site of incision of the artery. They were fixed in formalin and embedded in paraffin, and then hematoxylin-eosin and von Kossa staining were performed. The results were interpreted by an experienced pathologist who was blinded to the clinical data. Positive AMiC was defined as the presence of a von Kossa-stained area in the medial layers of the artery.

### 2.4. Statistical Analysis

The mean and standard deviations of continuous variables were calculated. To evaluate significant differences between continuous variables, Student's *t*-test and analysis of variance (ANOVA) were used for normally distributed variables; the Mann-Whitney *U* test and Kruskal-Wallis test were used for variables that were not normally distributed. The chi-square test and Fisher's exact test were used for comparisons of categorical variables. Multinomial logistic regression analysis was performed to investigate clinical factors related to AMiC. A *P* value < 0.05 was considered significant.

## 3. Results

### 3.1. Patient Characteristics

**Figure 1:**
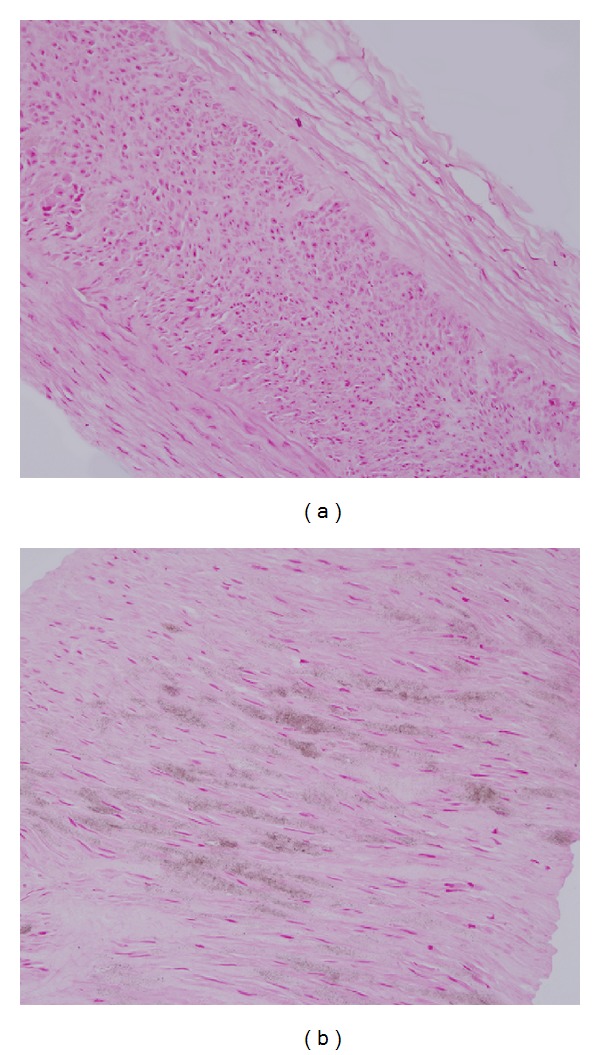
Histological findings of arterial microcalcification (AMiC). (a) AMiC negative, no staining (×200) (b) AMiC positive, positive staining with von Kossa stain (×200).

The mean age of the patients was 58.9 (range 19–83) years, and 52.4% were men. The most common cause of end-stage renal disease (ESRD) was diabetic nephropathy (53.7%); other causes included hypertensive nephropathy (20.2%) and chronic glomerulonephritis (16.7%). Sixty-seven (81.7%) of the patients had already started HD using catheters before vascular access surgery, and 15 (18.3%) had started HD after vascular access surgery. Six of 67 patients had received reoperation of previous vascular access. The mean HD duration for these patients was 2.5 months. AMiC was detected in 33 (40.2%) of the 82 patients and it was observed in only the medial layer of the arterial wall and not seen in the intimal layer (Figure 1).

### 3.2. Comparison of Clinical Characteristics according to Arterial Microcalcification

The clinical characteristics of patients with and without AMiC are summarized in Table 1. The mean age of patients with AMiC was higher than that of patients without AMiC (64.8 ± 10.1 versus 54.8 ± 15.9, *P* = 0.002). The patients with AMiC had a higher incidence of diabetes: 28 (84.8%) of 33 patients with AMiC had diabetes compared with 16 (32.7%) of 49 patients without AMiC (*P* = 0.001). There were no significant differences in whether or not to start HD before vascular access surgery and HD duration. The history of medication with calcium phosphate binder, noncalcium phosphate binder, vitamin D, and lipid-lowering agents also did not differ significantly between the two groups.

### 3.3. Comparison of Laboratory Characteristics according to Arterial Microcalcification

Table 2 summarizes the laboratory characteristics of patients with and without AMiC. The mean hemoglobin values and iron status including serum iron, ferritin, total iron-binding capacity, and TSAT did not differ between the patients with and without AMiC. The serum levels of albumin were higher in AMiC-negative patients than in AMiC-positive patients (3.6 ± 0.6 versus 3.3 ± 0.5, *P* = 0.035). The serum levels of total cholesterol were higher in AMiC-negative patients than in AMiC-positive patients (171.6 ± 46.6 versus 147.2 ± 40.5, *P* = 0.017). There were no significant differences between the two groups in serum levels of calcium, phosphate, and CaxP. Mean levels of serum intact parathyroid hormone (iPTH) were lower in AMiC-positive patients than in AMiC-negative patients (145.9 ± 108.9 versus 242.8 ± 167.8, *P* = 0.005). The mean EHRI was higher in AMiC-positive patients than in AMiC-negative patients (17.7 ± 11.8 versus 12.0 ± 13.9, *P* = 0.049).

### 3.4. Relationship between ESA Hyporesponsiveness and Arterial Microcalcification

We analyzed the correlation between ESA hyporesponsiveness and the presence of AMiC. The patients were divided into tertiles according to their EHRI values, as in our previous study [7]. The mean EHRI values for each tertile were 3.3 ± 1.7 (T1), 10.2 ± 3.3 (T2), and 29.8 ± 11.8 (T3).  Table 3 summarizes the clinical and laboratory characteristics according to the EHRI tertiles. There were no significant differences in the clinical and laboratory characteristics between the three groups except BMI and mean hemoglobin levels. Sixteen (59.3%) of 27 patients in T3 for EHRI were AMiC positive compared with 6 (21.4%) of 28 patients in T1 for EHRI (*P* = 0.017) (Table 4). When ESA hyporesponsiveness was defined, according to KDOQI guidelines, as more than 300 IU/kg per week recombinant human erythropoietin [4], AMiC was observed more frequently in patients with ESA hyporesponsiveness, but this was not statistically significant (*P* = 0.086) (Table 4). In the multivariate logistic regression analysis, the presence of diabetes and high EHRI values also showed a significant association with AMiC in HD patients (Table 5). There was no significant difference in EHRI tertiles between diabetes and nondiabetes, but serum iPTH levels were lower in patient with diabetes than in patients without diabetes (165.6 ± 111.6 versus 246.0 ± 182.5, *P* = 0.022).

## 4. Discussion

This study is derived from an improved understanding of the mechanisms common to ESA hyporesponsiveness and vascular calcification. The uremic state is characterized by increased oxidative stress and it is also related to the inflammatory conditions. We previously reported that inflammation plays a key role in the ESA hyporesponsiveness of HD patients who have sufficient iron [7]. Inflammation contributes to ESA hyporesponsiveness via proinflammatory cytokines such as IL-6, which antagonize the action of endogenous and exogenous erythropoietin by directly inhibiting erythroid progenitor cells and by the disruption of iron metabolism [13].

The mechanism of vascular calcification has been studied for decades, and growing evidence now suggests that vascular calcification is not the simple precipitation of calcium and phosphate but is considered to be a highly regulated pathological process that resembles osteogenesis [14]. Based on previous studies, a key step in vascular calcification appears to be differentiation or transformation of vascular smooth muscle cells into an osteoblastic/chondrocytic phenotype [8, 15]. There are multiple factors that induce this transformation, and oxidative stress and inflammation are considered to be among them. The contribution of oxidative stress and inflammation to the pathogenesis of vascular calcification has been described recently [10, 11]. Oxidative stress such as reactive oxygen species and inflammatory cytokines trigger molecular mechanisms that induce osteochondrogenesis and result in vascular calcification [11, 16–18]. Therefore, we hypothesized that vascular calcification may be associated with ESA hyporesponsiveness in HD patients.

In this study, we confirmed the presence of AMiC in 33 of 82 patients with CKD. The mean age of patients with AMiC was greater than that of patients without AMiC and patients with diabetes had a higher incidence of AMiC than patients without diabetes. Diabetes and older age are well-known risk factors for vascular calcification in CKD [9]. Hyperglycemia may influence vascular calcification through various mechanisms, including activation of vascular bone morphogenetic protein signaling, oxidative stress, and endothelial dysfunction [15]. Meanwhile, patients with AMiC had lower levels of serum albumin and total cholesterol compared with patients without AMiC. Serum albumin has been the most common nutritional marker in CRF patients. Therefore, these results suggest that there is an association between malnutrition and AMiC. This relationship can be explained on the basis of inflammation. Several reports have suggested the existence of a syndrome consisting of malnutrition, inflammation, and atherosclerosis (MIA syndrome) in some patients with CRF [19, 20]. Inflammation plays a significant role in causing hypoalbuminemia in CRF patients. Proinflammatory cytokines cause malnutrition by stimulating protein catabolism and by reducing albumin synthesis [19].

Patients with AMiC had lower levels of iPTH compared with patients without AMiC. Although there is still controversy, these results are consistent with those of several previous studies suggesting that low-turnover bone disease with low iPTH levels is more likely to be associated with vascular calcification [2, 21]. Finally, vascular calcification was more frequent in patients with ESA hyporesponsiveness. This result was confirmed in multivariate analysis, which showed that a high EHRI value was independently associated with AMiC. In this study, we do not perform the analysis of inflammatory markers such as IL-6, because our previous study has shown that patients with high EHRI value had significantly higher serum IL-6 levels than patients with low EHRI value [7].

On the other hand, abnormal mineral metabolism has been recognized as another risk factor for vascular calcification in CKD patients. Hyperphosphatemia and elevated serum CaxP have been associated with vascular calcification in CKD patients [8, 9]. Several studies have demonstrated that the use of calcium-containing phosphate binders increases vascular calcification [22]. However, in our study, serum phosphorus level and CaxP did not differ between patients with and without AMiC. There was also no significant difference in use of calcium-containing phosphate binder and vitamin D between the two groups. These results may reflect that abnormal mineral metabolism is not entirely responsible for vascular calcification in CKD and suggest that factors other than the serum levels of calcium and phosphorus play an important role in the development of vascular calcification. In addition, we have included the patients who received vascular access surgery to get vascular tissue and it has resulted in short dialysis duration. The effect of factors such as dialysis adequacy, uremia, calcium, and phosphorus on ESA hyporesponsiveness or AMiC may be underestimated by short dialysis duration.

## 5. Conclusions

Vascular calcification in patients with CKD is a significant problem and is associated with cardiovascular mortality. Therefore, investigation of factors related to vascular calcification is important. We found that in addition to the previously well-known risk factors of abnormal mineral metabolism and diabetes, resistance to ESAs may be associated with vascular calcification in patients with CKD.

## Figures and Tables

**Table 1:** Comparison of clinical parameters according to arterial microcalcification.

Clinical parameters	All patients (*n* = 82) number (%)	AMiC-negative (*n* = 49) number (%)	AMiC-positive (*n* = 33) number (%)	*P*
Age (years)^1^				
Mean ± SD	58.9 ± 14.6	54.8 ± 15.9	64.8 ± 10.1	**0.002**
Sex				
Men	43 (52.4)	25 (51.0)	18 (54.5)	0.754
Women	39 (47.6)	24 (49.0)	15 (45.5)	
BMI (kg/m^2^)^2^				
Mean ± SD	23.5 ± 3.3	23.4 ± 3.4	23.5 ± 3.3	0.863
Chronic kidney disease				
Diabetes	44 (53.7)	16 (32.7)	28 (84.8)	**0.001**
Nondiabetes	38 (46.3)	33 (67.3)	5 (15.2)	
Dialysis prior to vascular access creation	67 (81.7)	39 (79.6)	28 (84.8)	0.546
HD duration before Op^1^				
Mean ± SD (months)	2.5 ± 7.8	1.7 ± 5.1	3.5 ± 10.6	0.304
Kt/V^2^	1.42 ± 0.25	1.41 ± 0.22	1.43 ± 0.28	0.770
Residual renal function^1^ (mL/min)	4.92 ± 4.62	4.25 ± 4.05	5.90 ± 5.30	0.114
Phosphate binder				
Calcium	57 (69.5)	32 (65.3)	25 (75.8)	0.313
Noncalcium	14 (17.1)	8 (16.3)	6 (18.2)	0.827
Vitamin D	19 (23.2)	14 (28.6)	5 (15.2)	0.158
Lipid-lowering agents	27 (32.9)	14 (28.6)	13 (39.3)	0.306

AMiC: arterial microcalcification; BMI: body mass index; HD: hemodialysis; Op: operation for vascular access.

^1^These parameters were compared using Mann-Whitney *U* test and ^2^these parameters were compared using Student's *t*-test.

**Table 2:** Comparison of laboratory parameters according to arterial microcalcification.

Laboratory parameters	All patients (*n* = 82) number (%)	AMiC-negative (*n* = 49) number (%)	AMiC-positive (*n* = 33) number (%)	*P*
Hemoglobin (g/dL)	9.8 ± 0.7	9.9 ± 0.8	9.8 ± 0.6	0.403
WBC count (/mm^3^)	6,869 ± 1,844	7,011 ± 2,010	6,660 ± 1,571	0.402
Neutrophil count (/mm^3^)	4,533 ± 1,581	4,626 ± 1,751	4,396 ± 1,302	0.523
Iron (ug/dL)	68.8 ± 27.8	71.2 ± 28.9	65.4 ± 26.1	0.357
Ferritin (ng/mL)^1^	339.5 ± 309.7	347.1 ± 286.7	328.1 ± 345.3	0.787
TIBC (ug/dL)	208.6 ± 53.3	212.3 ± 54.1	203.0 ± 52.7	0.444
TSAT (%)^1^	33.4 ± 12.1	33.8 ± 12.1	32.8 ± 12.2	0.715
Albumin (g/dL)	3.5 ± 0.5	3.6 ± 0.6	3.3 ± 0.5	**0.035**
CRP (mg/dL)^1^	0.85 ± 1.53	0.78 ± 1.31	0.95 ± 1.81	0.637
T. cholesterol (mg/dL)^1^	161.8 ± 45.6	171.6 ± 46.6	147.2 ± 40.5	**0.017**
Triglyceride (mg/dL)^1^	165.4 ± 164.9	174.8±201.3	151.6 ± 87.7	0.535
Calcium (mg/dL)^1^	8.3 ± 1.0	8.1 ± 0.8	8.0 ± 0.7	0.354
Phosphate (mg/dL)	4.6 ± 1.0	4.5 ± 0.8	4.7 ± 1.2	0.532
CaxP product	38.3 ± 9.6	38.0 ± 7.7	38.8 ± 11.9	0.738
ALP (U/L)^1^	232.8 ± 113.8	244.4 ± 128.6	216.1 ± 86.9	0.241
nPCR (g/kg/d)	0.771 ± 0.283	0.767 ± 0.305	0.776 ± 0.258	0.890
iPTH (pg/mL)^1^	203.3 ± 153.6	242.8 ± 167.8	145.9 ± 108.9	**0.005**
EHRI^1^	14.3 ± 13.3	12.0 ± 13.9	17.7 ± 11.8	**0.049**

AMiC: arterial microcalcification; WBC: white blood cell; TIBC: total iron-binding capacity; TSAT: transferrin saturation; CRP: C-reactive protein; T. cholesterol: total cholesterol; CaxP product: calcium-phosphorus product; ALP: alkaline phosphatase; nPCR: normalized protein catabolic rate; iPTH: intact parathyroid hormone; EHRI: ESA hyporesponsiveness index.

^1^These parameters were compared using Mann-Whitney *U* test and the other parameters were compared using Student's *t*-test.

**Table 3:** Comparison of clinical and laboratory parameters according to EHRI tertiles.

Clinical parameters	EHRI tertiles			*P*
	T1 (*N* = 28)	T2 (*N* = 27)	T3 (*N* = 27)	
Age (years)^1^				0.345
Mean ± SD	57.7 ± 12.7	56.7 ± 17.5	62.1 ± 12.9	
Sex				
Men	19 (67.8)	14 (51.8)	10 (37.0)	0.073
Women	9 (32.2)	13 (48.2)	17 (63.0)	
BMI (kg/m^2^)				
Mean ± SD	23.7 ± 3.3	24.5 ± 3.5	22.1 ± 2.7	0.027*
Chronic kidney disease				
Diabetes	14 (50.0)	12 (44.4)	18 (66.7)	0.233
Nondiabetes	14 (50.0)	15 (55.6)	9 (33.3)	
Kt/V	1.38 ± 0.21	1.38 ± 0.30	1.48 ± 0.21	0.239
Hemoglobin (g/dL)	10.1 ± 0.8	9.9 ± 0.6	9.5 ± 0.5	**0.001**
WBC count (/mm^3^)	7,241 ± 1,965	6,710 ± 1,412	6,643 ± 1,844	0.423
Iron (ug/dL)	71.2 ± 27.2	72.6 ± 22.7	62.5 ± 32.4	0.352
Ferritin (ng/mL)^1^	359.1 ± 273.0	356.8 ± 388.4	301.7 ± 261.2	0.745
TSAT (%)^1^	33.4 ± 11.6	34.7 ± 11.2	32.0 ± 13.5	0.703
Albumin (g/dL)	3.6 ± 0.5	3.5 ± 0.5	3.3 ± 0.5	0.089
CRP (mg/dL)^1^	0.63 ± 1.16	0.69 ± 0.84	1.22 ± 2.20	0.303
T. cholesterol (mg/dL)^1^	165.9 ± 28.6	167.0 ± 44.0	152.3 ± 59.6	0.420
Calcium (mg/dL)^1^	8.1 ± 0.8	8.1 ± 0.7	8.1 ± 0.6	0.992
Phosphate (mg/dL)	4.9 ± 0.9	5.0 ± 0.9	4.4 ± 1.1	0.053
nPCR (g/kg/d)	0.785 ± 0.297	0.819 ± 0.286	0.713 ± 0.269	0.394
iPTH (pg/mL)^1^	249.2 ± 145.6	190.2 ± 161.8	170.6 ± 147.4	0.148

*The difference was shown between T2 and T3.

^1^These parameters were compared using Kruskal-Wallis test and the other parameters were compared using ANOVA.

EHRI: ESA hyporesponsiveness index; BMI: body mass index; WBC: white blood cell; TSAT: transferrin saturation; CRP: C-reactive protein; T. cholesterol: total cholesterol; nPCR: normalized protein catabolic rate; iPTH: intact parathyroid hormone.

**Table 4:** Arterial microcalcification according to EHRI values categorized into tertiles.

Arterial microcalcification	EHRI tertiles (mean ± SD)			*P* value
	T1 (*N* = 28) (3.3 ± 1.7)	T2 (*N* = 27) (10.2 ± 3.3)	T3 (*N* = 27) (29.8 ± 11.8)	
AMiC negative	22 (78.6)	16 (59.3)	11 (40.7)	**0.017**
AMiC positive	6 (21.4)	11 (40.7)	16 (59.3)	

	IU/kg/week recombinant human erythropoietin			
	≤300 (*N* = 71)	>300 (*N* = 11)		

AMiC negative	45 (63.3)	4 (36.4)		0.086
AMiC positive	26 (36.6)	7 (63.6)		

AMiC: arterial microcalcification; EHRI: ESA hyporesponsiveness index.

**Table 5:** Multivariate logistic regression analysis of arterial microcalcification^1^.

Parameters	Beta	Odds ratio	95% CI		*P* value
			Lower	Upper	
iPTH	−0.004	0.996	0.992	1.001	0.115
Diabetes	2.489	12.044	3.508	41.350	**0.001**
EHRI (T3)	1.479	4.390	1.053	18.306	**0.038**

^1^The reference category is AMiC-negative groups.
